# 2D Analysis of Gold Weight Implantation Surgery Results in Paralytic Lagophthalmos

**DOI:** 10.14744/bej.2021.95866

**Published:** 2021-09-27

**Authors:** Kubra Serefoğlu Cabuk, Gamze Ozturk Karabulut, Korhan Fazil, Senay Asik Nacaroglu, Zehra Karaagac Gunaydin, Muhittin Taskapili

**Affiliations:** 1. Department of Ophthalmology, University of Health Sciences, Beyoglu Eye Training and Research Hospital, Istanbul, Turkey; 2. Department of Ophthalmology, Dunyagoz Hospital, Istanbul, Turkey

**Keywords:** Gold weight, image analysis, ImageJ, paralytic lagophthalmos

## Abstract

**Objectives:**

Gold weight implantation in the upper eyelid is a frequently performed treatment for paralytic lagophthalmos to prevent corneal exposure. A margin reflex distance of -1 and -2 (MRD1, MRD2), the palpebral fissure height (PFH), and the vertical lagophthalmos (LV) are 1-dimensional (1D) measurements used in follow-up. Because the exposure area is 2-dimensional (2D), this study was designed to investigate the results using both 1D and 2D analysis.

**Methods:**

Ten patients who underwent pretarsal suborbicularis oculi gold weight implantation were included in the study. Photographs were taken with a digital camera and the images were analyzed using ImageJ software (US National Institutes of Health, Bethesda, MD, USA). The lagophthalmos area (LA) and ocular surface area (OSA) were measured in 2D in addition to the MRD1, MRD2, PFH, LV. Preoperative and postoperative values were compared using the Wilcoxon signed-rank test. Associations between parameters were evaluated using Spearman's correlation analysis.

**Results:**

The mean age of the patients (7 male, 3 female) was 39.6±16.4 years (range: 14–60 years). The mean implant weight was 1.46 g (0.8–1.6 g). There were significant reductions in the MRD1, MRD2, PFH, OSA, LV, and LA values after surgery (p<0.05). The weight of the gold implant had a strong correlation with the PFH, OSA, MRD1, and MRD2, but not the LV or LA, preoperatively. The OSA was strongly correlated with the MRD1, PFH, and the implant weight, but not the MRD2. The LA was strongly correlated with the LV, preoperatively. In the postoperative period, the OSA was strongly correlated with the PFH and the MRD2 but not the MRD1, while the LA was strongly correlated with the LV, MRD1, and the PFH.

**Conclusion:**

It is easy to obtain 2D measurements using digital image analysis software, and they proved to be accurate and correlated strongly with 1D measurements. The OSA and LA measurements were significantly lower following upper eyelid gold weight implantation. The PFH and LV were compatible with the OSA and LA, preoperatively.

## Introduction

Complications ranging from mild punctate epitheliopathy to refractory corneal ulcers, keratitis, corneal scars, and even perforation may occur in the case of lagophthalmos after facial palsy. To prevent these sight-threatening consequences, it is crucial to manage paralytic lagophthalmos from early-onset. The main aim of the therapy is to overcome lagophthalmos and restore ocular surface area (OSA) moisture (1). Ocular lubricants, nightly eyelid taping, temporary tarsorrhaphy, mullerectomy, levator muscle lengthening, lower eyelid tightening, suborbicularis oculi fat pad lifting, midface lifting, temporalis muscle transfer, upper eyelid hyaluronic acid injection and gold weight implantation are the choices of treatments (2-10). The treatment strategy is decided with regards to the severity of corneal disease and predicted facial palsy recovery time (1).

Upper eyelid pretarsal gold weight implantation is a frequently preferred treatment because of its effectiveness and reversibility (3, 4). The margin reflex distance 1 and 2 (MRD1 and MRD2), palpebral fissure height (PFH), the amount of vertical lagophthalmos (LV) are the 1D measurements used in the follow-up. We could not find a study presenting the upper eyelid gold weight results in 2D.

Because the exposure in lagophthalmos is 2D, we aimed to measure the areas together with the vertical distances by an image analysis software and investigated the correlation between them.

## Methods

The files and photos of the patients who underwent upper eyelid gold weight implantation for paralytic lagophthalmos between January 2017 and July 2019 in a tertiary care hospital were included in the study. Written informed consent was obtained from all patients. Patients lacking pre or post-operative photos and/or inadequate file content excluded from the study. Ethical approval was received from the Clinical Ethical Committee of the University of Health Sciences Haseki Training and Research Hospital (date: 21.10.2020, no: 2020-181). The study was conducted following the Declaration of Helsinki. Patients lacking photographic data, having lower eyelid surgery simultaneously with gold weight implantation were excluded from the study.

All of the patients underwent a full ophthalmic examination. Routine eyelid measurements including MRD1, MRD2, LV were noted on the file. Golden weight sizer plaques were attached on the central 1/3 pretarsal skin by double-sided tape. The weight causing minimal or no ptosis while reducing the lagophthalmos between 2 mm and 4 mm was chosen.

**Figure 1.**
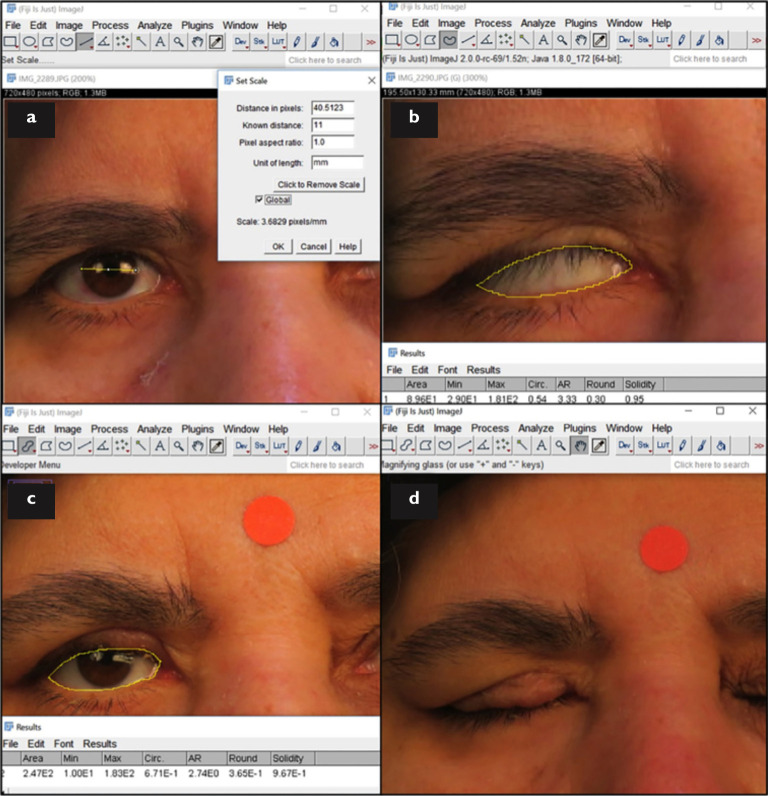
**(a)** Calibration acoording to radius of the iris. **(b)** Measuring the area of lagophthamos in preoperative eyelid closure. **(c)** Measuring the ocular surface area. **(d)** Postoperative eyelid closure.

After a 10 mm sticker was attached to the glabella, photographs were taken by a digital camera (Canon G3X, Canon Inc., Tokyo, Japan) in the same lightening condition of the photo-room facing the camera, and chin-up while detecting lagophthalmos. Images were transferred to the computer. The setting scale was done using a 10 mm glabellar sticker. Iris diameter of new photos measured and used as a caliper for the earlier photos without a sticker of the same patient. The MRD1 and MRD2, PFH, and the amount of LV is measured linearly, area of lagophthalmos (LA) and ocular surface (OSA) are measured 2D by ImageJ (Fig. 1). All measurements were repeated three times by the same doctor. Manual measurements were also recorded in pre-and post-operative visits and compared with digital equivalents.

The implants were made up of 99.9% pure gold in the shape of curved rectangular, fitting well on the tarsus with rounded corners and edges including three holes enabling suture pass. All of them were produced in the same center.

All of the surgeries performed by 2 surgeons under local anesthesia by the same technique. 20 mg/ml lidocaine hydroclorur and 0.0125 mg/ml epinephrine infiltrated after marking the incision site throughout the upper eyelid crease. Skin and pretarsal orbicularis oculi are incised and the pretarsal area was exposed until 1–2 mm to the eyelash margin. Gold implants are fixed to the central tarsus by 6.0 vicryl. The incision was closed separately by 3 pieces of 6.0 vicryl suture to form lid crease then continuously by 6.0 polypropylene suture.

All statistical analyses were performed with SPSS v. 22.0 (SPSS Inc., Chicago, IL). Descriptive statistics included mean values ± SD for normally distributed variables. Distributions of the variables were measured by Kolmogorov–Smirnov test. For quantitative analysis, Wilcoxon signed-rank test was used. Correlation between parameters was evaluated by Spearman’s correlation analysis. A P -value below 0.05 was considered statistically significant.

## Results

The mean age of 10 patients (7 male and 3 female) was 39.6±16.4 years (range, 14–60 years). The mean follow-up period was 113 days (ranging from 93 to 150 days). The mean implant weight was 1.46 g (0.8–1.6 g). Etiologies of facial palsy were cranial tumor (n=2), cerebellopontine angle tumor (n=1), motor vehicle accident (n=2), and Bell’s palsy (n=5). There were no intraoperative complications. Preoperative exposure keratopathies resolved in 2 weeks after surgery. Hyperemia and thickening of the skin were seen in 2 patients which were resolved using topical steroid ointments. Implant visibility occurred in 1 patient but neither surgical success nor patient satisfaction was effected so no additional surgery was needed. No extrusion was seen in any of the implants. Explantation was performed because of recovery in one patient after 2 years.

There was no significant difference between the manual and digital measurements of MRD1, MRD2, PFH, LV (p>0.05).

There were significant reductions in MRD1, PFH, lagophthalmos vertical, OSA, and lagophthalmos area 3 months after surgery (Table 1).

**Table 1.** Postoperative changes in the findings

	**Preoperative**	Postoperative	P-values
MRD1 (mm)	4±1	2.21±0.6*	0.012
MRD2 (mm)	7.6±1	7.6±1	0.28
Palpebral fissure height (mm)	11.6±1.7	8.7±1.3*	0.012
Palpebral fissure area (mm^2^)	203±36	147.5±18.4*	0.012
Lagophthalmos vertical (mm)	7,41±2.3	2.3±1.7*	0.012
Lagophthalmos area (mm^2^)	132.5±38.6	33.2±29*	0.012

MRD1: Margin reflex distance 1; MRD2: Margin reflex distance 2. *p<0.05, statistically significant.

Correlations between pre-and postoperative measurements are shown in Tables 2 and 3.

**Table 2.** Preoperative correlation between measurements

		Weight	MRD1	MRD2	PFH	PFA
Weight (gr)	r	1	.724*	.781*	.905**	.732*
	p		.042	.022	.002	.039
MRD1 (mm)	r	.724*	1	.383	.831*	.970**
	p	.042		.349	.011	.000
MRD2 (mm)	r	.781*	.383	1	.832*	.500
	p	.022	.349		.010	.207
PFH (mm)	r	.905**	.831*	.832*	1	.883**
	p	.002	.011	.010		.004
PFA (mm2)	r	.732*	.970**	.500	.883**	1
	p	.039	.000	.207	.004	

Spearman’s correlation analysis, r: correalation coefficient, **p<0.001, *p<0.05. MRD: Margin reflex distance; PFH: Palpebral fissure height; PFA: Palpebral fissure area.

**Table 3.** Postoperative correlation between measurements

		Weight	MRD1	MRD2	PFH	PFA
Weight (gr)	r	1	.369	.683	.650	.703
	p		.368	.062	.081	.052
MRD1 (mm)	r	.369	1	.415	.786*	.624
	p	.368		.307	.021	.098
MRD2 (mm)	r	.683	.415	1	.889**	.809*
	p	.062	.307		.003	.015
PFH (mm)	r	.650	.786*	.889**	1	.864**
	p	.081	.021	.003		.006
PFA (mm2)	r	.703	.624	.809*	.864**	1
	p	.052	.098	.015	.006	

Spearman’s correlation analysis, r: correalation coefficient, **p<0.001, *p<0.05. MRD: Margin reflex distance; PFH: Palpebral fissure height; PFA: Palpebral fissure area.

Preoperative LA was strongly correlated with preoperative LV (r=0.99, p=0.00). Postoperative LA was strongly correlated with postoperative LV (r=0.98, p=0.00), MRD1 (r=0.84, p=0.009) and PFH (r=0.78, p=0.009).

## Discussion

Complications ranging from corneal punctate keratopathy to permanent corneal opacification and even spontaneous corneal perforation may be seen as a result of corneal exposure emerging from facial palsy (10). So from the early onset of paralytic lagophthalmos, it is very important to protect the ocular surface and restore lubrication (1, 12). Nightly eyelid taping, temporary tarsorrhaphy and fillers are the reversible choices of treatment. Lid loading, mullerectomy, levator muscle lengthening, lateral tarsal strip, suborbicularis oculi fat lift, midface lift, temporalis muscle transfer are mostly saved for long-lasting cases (1).

Upper eyelid gold weight implantation in the case of paralytic lagophthalmos is a frequently preferred surgery because of its effectiveness and reversibility (4, 12). The weight enhances gravity to close the upper eyelid (13). Foreign body reaction, allergic reaction, permanent redness, astigmatism, entropion, eyelash ptosis, ptosis, inadequate improvement, implant visibility, bulging or migration of the implant and implant extrusion are the main complications (14). Even if it is shown in some studies that platinum implantation and/or post septal implantation lessen most of these side effects, pretarsal gold weight implantation still keeps its popularity (15).

In 1983 Rolando et al. described the measurement of the OSA using an enlarged photography image and calculator plotter (16). They presented an equation of “OSA (cm^2^) = 0.28 × PFH (cm) - 0.44”. Sotoyama et al. accepted the OSA as a part of the spheric area and used the equation of “OSA (cm^2^) = 3.05 × PFH (cm) - 0.39 ” to estimate it (17). He described the OSA in a nice schematic diagram. However, periorbital features vary according to age, sex and ethnicity so these equations are inadequate to measure the 2D data precisely (18-21).

With the development of digital imaging technology and analyzing software, measuring throughout the images became more accurately and quantitatively. ImageJ is a free and reliable digital image analysis software available from the National Institute of Health, Bethesda, Maryland, USA (http://rsb.info.nih.gov/ij). It has been used in several disciplines successfully (22-24). In periorbital regions, Cruz et al. used ImageJ in 1998 to assess upper eyelid contours quantitatively in Graves and congenital blepharoptosis (25). Nunes et al. found no significant difference between measurements done manually and digitally and thus concluded that results obtained by photogrammetry of digital images are as reliable as a direct measurement (26). In our study, there was no significant difference in manual and digital measurements, also.

Rajyalakshhmi et al. put forth that periorbital biometric measurements using ImageJ software were reproducible and repeatable (27). Zheng et al. exposed eyelid features and eyebrow position following CO_2_ Laser-assisted blepharoptosis surgery by digital image analysis more accurately and quantitatively and underlined the increase in OSA in addition to MRD1 after surgery (28). Tsai et al. presented an increase in OSA and improvement in ocular asymmetry after double eyelid blepharoplasty using ImageJ (29).

In paralytic lagophthalmos, Mancini et al. used ImageJ to present the effect of hyaluronic acid use in the upper eyelid (2). They showed a significant improvement in lagophthalmos but only by the 1D measurements. Significant improvement in lagophthalmos were evident in our study too.

Success criteria after upper eyelid gold weight implantation were defined as a minimum 50% reduction in lagophthalmos or to minimize LV between 2 mm and 4 mm without inducing >2 mm ptosis (30-32). Postoperative LV was 2.3±1.7 mm in our study and reduction in LV and LA were >50% except for one patient (54%) despite 1.6 g weight. No increment in the gold weight has been performed because of adequate symptom relief.

Ptosis was evident in all of our cases similar to the literature (12). In the consequent of this, MRD1, PFH, and OSA reduced significantly after surgery. Although ptosis was >2 mm in 2 patients, we did not reduce the weights of them because of remaining corneal exposure in one of them and patient desire in the other.

The weight of the golden implant was most strongly correlated with preoperative PFH, OSA, MRD1, and MRD2 but not with LV or lagophthalmos area.

Although preoperative weight decision is made considering LV and ptosis, we did not find a correlation between them (31, 33). This may be because of the lower case number.

OSA was most strongly correlated with MRD1, than with PFH and gold weight heaviness but not with MRD2 preoperatively. The upper eyelid curvature is steeper than the lower eyelid so mainly the upper eyelid area determines the OSA preoperatively. MRD1 overlaps the upper eyelid radius, therefore OSA is most correlated with MRD1 (17).

Postoperative OSA was strongly correlated with postoperative PFH and MRD2 but not with MRD1. Because of postoperative ptosis, the curvature of the upper eyelid gets shorter, then PFH mainly consists of MRD2. PFH should be used either pre or postoperatively to estimate OSA. However, to differentiate upper and lower eyelid behaviors, it is meaningful to use MRD1 and MRD2 in clinical practice.

The restrictions of this study are lower case numbers. Studies with higher case numbers with preoperative weight decisions by digital photograph analysis would further lighten the exact equations between weight and the 2D measurements.

Upper eyelid gold weight implantation is a very effective surgery in the treatment of paralytic lagophthalmos. Digital image analysis guides the oculoplastic surgeon to see the improvement after periocular surgeries while delivering the advantage of talking about the results with the patient. OSA and LA significantly reduced after upper eyelid gold weight implantation. PFH and LV were found to be compatible with OSA and LA, respectively, perioperatively.

## Disclosures

### Ethics Committee Approval:

University of Health Sciences Haseki Training and Research Hospital Clinical Research Ethics Committee, protocol number: 2020-181, Date: 21/10/2020.

### Peer-review:

Externally peer-reviewed.

## Conflict of Interest:

None declared.

### Authorship Contributions:

Involved in design and conduct of the study (KSC, GOK); preparation and review of the study (KSC, GOK, KF, SAN, MT); data collection (KSC, SAN, ZKG, MT); and statistical analysis (KSC, SAN).
